# Proposal of a new way to evaluate the external sphincter function prior male sling surgey

**DOI:** 10.1590/S1677-5538.IBJU.2018.0146

**Published:** 2019-04-01

**Authors:** Daniel Carlos Moser, Carlos Arturo Levi D'ancona, Brunno Raphael Iamashita Voris, Daniel Lahan, Kavina Jani, Gerard D. Henry

**Affiliations:** 1 Departamento de Cirurgia Urológica, Universidade Estadual de Campinas - UNICAMP, Campinas, SP, Brasil;; 2 Department of Urology, Ark-La-Tex Urology, Shreveport, Louisiana, USA

**Keywords:** Urinary Incontinence, Prostatectomy, Transurethral Resection of Prostate

## Abstract

**Objective:**

To propose a new way to objectively evaluate the external sphincter function prior to male sling surgery.

**Materials and Methods:**

We evaluated the pre-operative sphincter function throughout sphincter pressure at rest (SPAR) and sphincter pressure under contraction (SPUC) obtained throughout urethral profilometry profile (UPP) of 10 consecutive patients (age range, 54-79 years) treated with the retrourethral transobturator sling (RTS) for stress urinary incontinence (SUI) because of prostate surgery. The primary endpoint for surgery success rate was post-operative pad weight test. This was correlated to pre-operative pad test, RT, SPAR and SPUC. Post-operatively patients were classified as continent (no pad use) and those who still were incontinent.

**Results:**

Mean SPUC in the continent and incontinent group was respectively 188 + 8.8 (median 185.1, range 181 to 201) and 96.9 + 49.4 (median 109.9, range 35.6 to 163.6) (P = 0.008). Mean 24-hour pad test was 151 + 84.2gm (median 140, range 80 to 245) and 973 + 337.1gm (median 1940, range 550 to 1200) in post-operative continent and incontinent groups respectively (P = 0.008). The repositioning test (RT) was positive in all continent patients except one. The RT was also positive in three incontinence patients (false positive). In all post-operative continent patients SPUC was higher than 180cmH2O and pre-operative pad test was less than 245gm.

**Conclusions:**

SPUC seems to be a way for optimizing the sphincter evaluation as well to become a useful tool for patient selection prior to RTS surgery.

## INTRODUCTION

Retrourethral transobturator sling (RTS) is a functional, non-compressive and nonobstructive minimally invasive treatment for stress urinary incontinence (SUI). When the strict definition of continence of 0 pads / 24-hour is used, cure rates of 80% are reported as good results on selected patient cohorts (1–4). Nevertheless, a failure rate between 20% and 45% of this technique has been reported with no clearly defined reasons. Reasons for failure of the primary RTS might be related to incorrect sling placement technique, sling slippage, radiation therapy, presence of periurethral fibrosis, bladder neck contraction or incorrect patient selection (5). The key mechanism to RTS surgery seems to be a dynamic support of the urinary sphincter during stress by increasing the coaptive zone in the sphincter part of the urethra and the ideal candidates for sling placement seem to be those with good residual urinary sphincter function (5).

Some urologists think a preoperative cystoscopy to evaluate sphincter function seems reasonable for optimal selection of patients (6). The repositioning test (RT) is a method proposed to evaluate the sphincter function on a minimally invasive way (7). A positive RT consists of contractility with a coaptive zone of ≥ 1 cm during external sphincter voluntary contraction (7). It is our belief that a possible factor influencing the outcome of the RT could be its interpretation. This is a subjective, non-numeric test, and depends largely on surgeon experience with the test. This element has not been stressed in published articles concerning repositioning slings surgeries and in particular repositioning test reports. Urethral pressure profilometry (UPP) was first described by Brown-Wickham in 1969 and was the first method used for evaluating urethral function (including sphincter pressure) (8). It is not largely used in pre-operative evaluation to male sling surgery and is more used to measure the sphincter pressure at rest (SPAR) than the sphincter pressure under contraction (SPUC) (9).

This study is a preliminary report proposing the use of the SPUC as a new and objective way to evaluate of the external sphincter function prior RTS surgery.

## MATERIALS AND METHODS

### Study Group

Between April 2016 and April 2017 ten consecutive patients with median age 68.5 (54-79) and duration of incontinence of 88.3 ± 71.4 months had comprehensive incontinence workup done for stress urinary incontinence (SUI). Prior the sling surgery, retropubic radical prostatectomy (RRP) was performed in 4 (40%) patients, transurethral resection of the prostate (TURP) in 4 (40%) and RRP associated with salvage radiation therapy in 2 (20%). The incontinent assessment included the International Consultation on Incontinence Questionnaire - Short Form (ICIQ-SF), 24-hour pad test, urodynamics, urethroscopy and RT. Urodynamics was performed according to the International Continence Society (ICS) recommendations (10). During urodynamics the urethral pressure profilometry (UPP) was performed to evaluate sphincter function. Measurements of SPAR and SPUC were recorded (detailed description below). RT was performed during cystoscopy to evaluate urethral mobility and sphincter function as described by Rehder P (4, 11). All patients underwent a RTS surgery and the same assessment was repeated in the postoperative (except urodynamics). Postoperatively patients were divided in two groups: continent or incontinent. Definition of continence was no pad usage.

The time elapsed between prostate and sling surgery was greater than 26 months. The surgeries were performed by two experienced urologists according to the technique described by Redher and Gozzi (12). A polyvinylidene fluoride (PVDF) sling was used, which is a highly non-reactive thermoplastic fluoropolymer produced by the polymerization of vinylidene difluoride, Dynamesh-PMR™. Exclusion criteria included the presence of anastomotic or urethral strictures on cystoscopy, high glucose blood levels (glycosylated hemoglobin higher than 7.5%), and previously failed treatments for incontinence. Informed consent was obtained from all patients and ethical institutional review board approved the study.

### Sphincter pressure at rest and under contraction (SPAR and SPUC)

**Figure 1:**
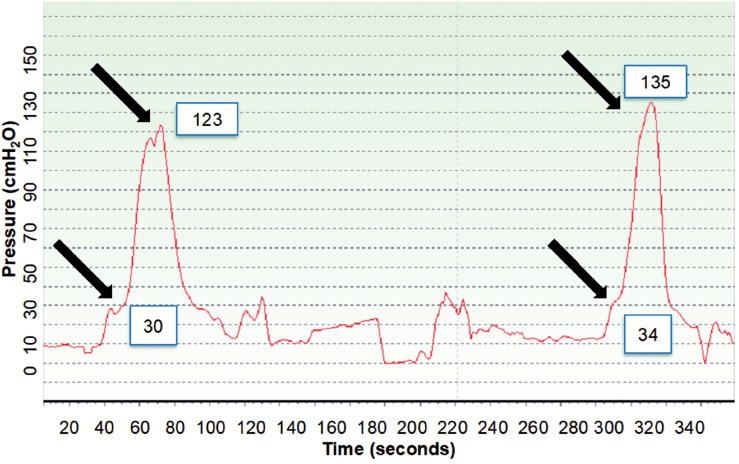
SPAR (bottom arrow) immediately before the voluntary contraction that leads to SPUC (top arrow).

The SPAR and SPUC evaluation were done according to the Brown-Wickham water perfusion method of urethral profilometry profile with a 10F catheter with four holes around the circumference, 5 cm distal of the tip (8). Transducers were zeroed to atmospheric pressure at the pubic symphysis level. The catheter was introduced into the bladder. The bladder was filled with 150 mL of normal saline solution at room temperature, and with the patient in the lying position the urethral catheter was manually withdrawn. The perfusion rate was 2 mL / min. The infusion and transducer lines were connected to the bladder catheter through a three-way tap to register initial bladder pressure. The catheter was withdrawn at 1 mm / s traction down the urethra and the pressure profile was recorded. The point of high pressure was considered the external sphincter localization. At this point the pressure was recorded as the SPAR. Then patients were asked to perform a pelvic floor contraction maneuver and the SPUC was recorded. This maneuver was repeated five times, with a three minutes interval and the medium value of the three highest SPUC were obtained for statistical analyses. Finally, the catheter was withdrawn until the holes around the circumference were clear of the external meatus (Figure-1)

### Statistical analysis

The analyses of results obtained from preoperative assessment (24h-pad test, RT, SPAR and SPUC) were performed using the two-tailed Mann-Whitney U test. A P-value < 0.05 was considered statistically significant. Statistical analysis was carried out using software SAS System for Windows (Statistical Analysis System), version 9.4. SAS Institute Inc, Cary, NC, US

## RESULTS

Median follow-up was 12 months (6–19). There were no major complications regarding sling implant. According to the aforementioned continence criteria the results were analyzed in two groups according to the postoperative 24h-pad test (primary endpoint). On this way 4 of 10 (40%) composed the continent group and 6 of 10 (60%) the incontinent one. The ICIQ-SF score in the preoperative in the continent and incontinent group were respectively 17.7 ± 1.2 and 18.3 ± 2.3 (p = 0.51). In the postoperative period this score in continent and incontinent groups turned respectively to zero and 14.6 ± 2.42 (p = 0.01). There was also no significant difference in preoperative urodynamic parameters between continent and incontinent groups.

The main results of this study are resume in Table-1. Pre-operatively 24-hour pad test in the continent group was 151 ± 84.2 gm (median 140, range 80 to 245) and in the incontinent group was 973 ± 337.1 gm (median 1940, range 550 to 1200) (p = 0.008). Mean SPAR in the continent and incontinent group were respectively 65.2 ± 22.5 cmH_2_0 (median 62.8, range 40.6 to 94.6) and 39.5 ± 12.9 (median 41.1, range 23 to 58) (p = 0.03). Mean SPUC in the continent and incontinent group were respectively 188 ± 8.8 cmH_2_0 (median 185.1, range 181 to 201) and 96.9 ± 49.4 cmH_2_0 (median 109.9, range 35.6 to 163.6) (p = 0.008). In all continent patients SPUC was higher than 180 cmH_2_0. The RT was positive in 3 / 4 continent patients and 3 / 6 in of the incontinence patients (false positive). The SPUC in false positive RT patients were 163.6, 120, and 100.6 cmH_2_0 respectively. RT was negative in no continent patient (false negative) and in 3 / 6 incontinent patients. All patients with low weight pad test (under 245 gm) presented with high pressure SPUC (over 180 cmH_2_0) and achieved complete continence. In the two patients with very low SPUC (patients #6 and #8) the repositioning test was negative and the pad test had high weight. Even in patients that did not achieve complete cures (SPUC < 180 cmH_2_0) there was a positive correlation between SPUC and postoperative pad test values. In the patient with the SPUC 163.6 cmH_2_O the pad test reduction was better compared to patients with SPUC 120 cmH_2_O or lesser (85% vs. 42-52% reduction) (Table-2).

**Table 1:** Pre and postoperative 24-h pad test, SPAR, SPUC and RT in postoperative continent and incontinent patients.

		24-h Pad test (gm)				
Patients		Preop	Postop	SPAR(cmH_2_O)	SPUC(cmH_2_O)	RT
**Continent**						
	#1	80	0	40.6	184.3	positive
	#2	200	0	67.3	181	negative
	#3	80	0	58.3	186	positive
	#4	245	0	94.6	201	positive
**Incontinent**						
	#5	740	100	58	163.6	positive
	#6	1200	570	27	35.6	negative
	#7	750	400	23	120	positive
	#8	1400	670	40.3	42.3	negative
	#9	550	320	42	100.6	positive
	#10	1200	600	47	119.3	negative

**Table 2:** SPUC and percentile of improvement.

SPUC (cmH_2_O)	Pad test reduction
≥ 180	100%
163.6	86%
120	50%
119.6	46%
100.6	42%
42.3	52%
35.6	52%

## DISCUSSION

This study is a preliminary report proposing the use of SPUC as an objective way to evaluate the external sphincter function prior to male sling surgery. Reasons for primary sling failure are still poorly understood and may be related to an inappropriate indication or technique (13). Patient selection is probably the most important factor related to sling surgery results but there is still not complete standardization on the selection methods used (14). An “ideal” patient to sling implant is described as a non-irradiated, with no previous urethral surgeries, only mild-to-moderate UI with a threshold of 200 gm on a 24-h pad test, cystoscopy should exclude concomitant urethral strictures / bladder neck contracture and the repositioning test should assure good urethral mobility and sphincter coaptation (15). Beside these statements papers still cannot explain why some “ideal” patients do not get completely dry and why some “no ideal” patients get dry. As demonstrated, the reported rate of RTS failure is 20% to 45.5% (13). To get these answers and consequently better results Rehder et al. presented a review explaining the potential mechanism of RTS in the therapy of post-prostatectomy UI (16). These and other authors agree that the key mechanism seems to be a dynamic support of the sphincter during stress by increasing the zone of coaption in the sphincter part of the urethra (5, 16). To the authors a preoperative evaluation of sphincter function appears to be an important aspect for optimal selection of patients. Other papers also highlight the importance of preoperative endoscopic evaluation whilst only pad usage is shown to be an independent predictor of success (2, 19, 20). In a single-center prospective study Bauer et al. reported 65 consecutive patients with SUI after radical prostatectomy submitted to the repositioning test. Preoperatively patients were classified as positive and negative RT. 53 patients (81.5%) showed preoperatively a positive RT and 12 patients (18.5%) a negative RT. After a follow-up of 12 months, patients with positive RT showed a cure rate (0 pads / day) of 83% and patients with a negative RT showed only a cure rate of 25%. A positive RT significantly correlated with cure in outcome (p < 0.001) (7). This ideal group with SUI to be treated with repositioning slings includes patients with only mild-to-moderate urinary incontinence, no nocturnal urinary incontinence, no prior history of radiotherapy and positive RT (6).

In our opinion, RT is extremely observer dependent. The correct classification of positive or negative test is completely visual and may vary between observers. Therefore, the RT is a subjective and non-numeric test. It is also hard to compare RT results and consequently preoperative characteristics between different cohorts. This test seems to be very useful in the selection but its subjectivity may be a barrier to a widely usage. In our cohort false positive rates in RT were found in 30% of the patients, which may be a possible explanation to failure rates on “ideal“ candidates to RTS. The RT was positive in three patients that did not achieve complete continence. In these three patients, SPUC were respectively 163.6, 120 and 100.6 cmH_2_O demonstrating that they presented contraction but not enough to get continence after sling implantation. Nowadays pad test seems to be one of the best-studied and accepted predictors of success. Collado Serra et al. demonstrated that preoperative 24-hour pad weight correlated inversely with the outcome (odds ratio 0.996), with a 0.4% decrease in cure rate for each 1g increase in the preoperative 24-hour pad weight (21). Rehder et al. also described a 1-year postoperative success rate (defined as 1-2 pad per day and > 50% reduction in pad use) with the Advance® sling of 94% (107 of 114 patients) (22). In our study, all patients that presented with SPUC values higher than 180 cmH2O had low weight pad test (under 245 gm) demonstrating good correlation between the two methods. The big question for pad test usage only is if there are patients with higher pad test volumes and good residual sphincter function that could be included on sling protocols. One interesting paper, Malik et al. reported the variability of the pad test according to the amount of physical activity performed by the patient on the day of collection. According to the author, as higher the degree of physical activity on the day of collection the higher will be the pad test weight (23). This aspect reinforce our hypotheses that lower values in the pad test can lead to false “ideal” patients and the objective evaluation of sphincter function could help selecting patients to RTS surgery. To the best of our knowledge, there is no report using the SPAR and SPUC to predict success in RTS surgery. Commiter et al. studied the correlation among maximal urethral closure pressure, retrograde leak point pressure, and abdominal leak point pressure in men with postprostatectomy stress incontinence (9). All these pressure measurements are different from the measurements performed in this study. On this preliminary report, the SPAR and SPUC (especially SPUC) presented good association with sling surgery success.

Possible criticism to this preliminary report are the different etiologies for incontinence with a mixed cohort of post TURP and post PRR patients without details on radiotherapy and high values of pad test patients. Our arguments for mixed cohort are that our main objective was to evaluate the sphincter function independent of the etiology of incontinence. Kretschmer et al. published long-term outcome of the RTS after TURP in a cohort of 15 patients with a median follow-up of 70 months (range, 18-83 months) and mean daily pad usage was 1.8 ± 2.1 pads. Cure rate was 46.7%, and cure-and-improved rate was 60.0% (2). The authors concluded that AdVance® and AdVanceXP® implantation can be performed effectively and safely in men suffering from SUI after TURP. However, long-term success rates seem to be lower compared to SUI after radical prostatectomy and patients should be counseled accordingly. In our cohort the separate assessment of post-TUR patients has showed that the degree of sphincter injury is more important than the etiology of incontinence when deciding whether or not to include a patient in a sling protocol. Two of our TURP patients that presented low volume of pad test and high SPUC in the preoperative period values were cured (patients #1 and #2). One of them presented SPUC of 163 cmH2O and had a reduction of 86% in the volume of losses using only 1pad / day (patient #3). The last one (patient #4) presented with a high pad test with low SPUC value evolving with great reduction of the pad test but still incontinent (Table-1). This is in line with the literature and we believe that the results of the RTS post TURP can be optimized with a better preoperative evaluation of the sphincter function. Before criticisms related to the large volumes of pad test of patients submitted to sling surgery it is important to understand that there are economic disparities on the planet we we all live. In our country more than half of the population does not have medical insurance and for the patients in this cohort the access to the AUS was completely out of possibilities. In Brazil, the final cost of an AUS is US$ 25.000.00 versus US$ 12.000.00 (also current coin relation is 3.4 R$ = 1 US$) and for these patients the sling surgery is frequently the only hope to improve the incontinence rates. In cases that the gold standard is not possible even a reduction in pad usage represents a huge impact on patients quality of life. It is also important to note that in this study even among patients with large loss volumes in preoperative pad test a reduction of 50% in almost of them was achieved. The large pad test weight in some of the subjects enrolled in this protocol was also important to better understand the sphincter function in these particular situations. Every patient with high pad test weight enrolled in this protocol was aware that the AUS was the gold standard to fix their problem but given the circumstances (impossibility to get an AUS implant due high costs) all of them fully agreed to undergo to the sling surgery even knowing that they would not be cured but glad with the perspective that they would need to buy and change fewer diapers per day.

The main limitation to our study is the small population of patients. Once this is a proposal of new way to measure the sphincter function before sling surgery we do not have comparison studies to confirm our data. More patients are just enrolled in our protocol and we hope to show more data soon. Other centers reproducing the technique and comparing to sling surgery results are welcome. Another important limitation is related to the technique employed in the measurements of SPUC. The ICS published standards on urethral pressure measurement in 2002, but internal and external consistency, retest reliability, and sensitivity to change have never been quantified (24). Also, there is no agreed-upon approach to ensure high quality (reliable and valid) urodynamic testing at maximum urethral closure pressure and during pelvic floor muscle contraction (25). In spite of these limitations, we believe that an objective sphincter pressure cut off value could be an additional tool to help both surgeons and patients to decide what surgical method to choose to fix incontinence in men.

## CONCLUSIONS

This is a preliminary report proposing the use of SPUC as objective evaluation of the external sphincter function prior male sling surgery. SPUC needs to be reproduced in larger cohorts to be validated and standardized but seems to be a way for optimizing the sphincter evaluation as well to become a useful tool for patient selection to RTS surgery.
